# miR-769-5p suppressed cell proliferation, migration and invasion by targeting TGFBR1 in non-small cell lung carcinoma

**DOI:** 10.18632/oncotarget.23060

**Published:** 2017-12-08

**Authors:** Zhao Yang, Jin He, Peng Gao, Yi Niu, Jie Zhang, Lei Wang, Meiyue Liu, Xiaomei Wei, Chunling Liu, Chao Zhang, Wei Wang, Jiayi Du, Hongmin Li, Wanning Hu, Guogui Sun

**Affiliations:** ^1^ Department of Radiation Oncology, North China University of Science and Technology Affiliated People's Hospital, Tangshan, China; ^2^ Department of Hepatobiliary Tumor, Tianjin Medical University Cancer Institute and Hospital, National Clinical Research Cencer, Tianjin, China; ^3^ Department of Pathology, North China University of Science and Technology Affiliated People's Hospital, Tangshan, China

**Keywords:** lung cancer, microRNAs, invasion and metastasis, gene therapy

## Abstract

MicroRNAs (miRNAs) are key regulators of multiple cancers, including non-small cell lung carcinoma (NSCLC). The aim of this study was to determine the expression pattern of miR-769-5p in NSCLC and to investigate its biological role during tumorigenesis. We showed that miR-769-5p was significantly downregulated and predicted poor prognosis in NSCLC compared with corresponding normal tissues. We then investigated its function and found that miR-769-5p significantly inhibited cell proliferation, migration and invasion *in vitro* and reduced tumor growth and metastasis *in vivo*. Furthermore, we explored the molecular mechanisms by which miR-769-5p contributes to NSCLC suppression and identified TGFBR1 as a direct target gene of miR-769-5p. Finally, we showed that TGFBR1 had opposite effects to those of miR-769-5p on lung cancer cells, suggesting that miR-769-5p might inhibit lung tumorigenesis by silencing TGFBR1. Taken together, our results demonstrated that miR-769-5p plays a pivotal role in NSCLC by inhibiting cell proliferation, migration and invasion by targeting TGFBR1.

## INTRODUCTION

Lung cancer is one of the most frequently diagnosed cancers and is the leading cause of cancer-associated death in both men and women worldwide. There were an estimated 1.80 million new cases in 2012; extrapolating from a 2012 International Agency for Research on Cancer (IARC) risk assessment [1], this disease kills approximately 1.59 million people per year globally, and this trend is expected to continue until 2030. Approximately 85% of lung cancers are histopathologically classified as non-small cell lung carcinomas (NSCLC) [2]. Treatment advances have been made in surgery and chemotherapy [3], but the 5-year overall survival (OS) rate is only 16% for all stages [4]. The initiation and development of NSCLC is attributed to aberrant expression of proto-oncogenes and tumor-suppressive genes, which leads to tumor cell growth, metastasis, and eventually tumor progression. At the molecular level, lung cancer arises from a series of genetic and epigenetic alterations that inactivate tumor suppressor genes and activate oncogenes. However, the basic mechanisms underlying lung cancer initiation and progression remain largely unknown. A greater understanding of the molecular mechanisms underlying carcinogenesis, progression and drug resistance in lung cancer would be helpful in improving diagnosis, therapy and prevention.

MicroRNAs (miRNA) are a class of small noncoding RNAs of 18–24 nucleotides that bind to partially complementary recognition sequences in mRNAs, causing either degradation or inhibition of translation, thus effectively silencing their mRNA targets [5]. It is estimated that approximately one-third to one-half of human genes are directly regulated by miRNAs, with each miRNA predicted to target several hundred transcripts, making miRNAs one of the largest families of gene regulators [6]. MiRNAs binding to the 3′-untranslated region (3′UTR) of target mRNAs leads to translational repression or degradation of the mRNA. MiRNAs may be new therapeutic strategies for cancers, as they can act as tumor suppressors or oncogenes depending on their target mRNAs [7–9]. Furthermore, many recent studies have shown that some miRNAs cooperatively control a variety of biological processes, including cell proliferation, migration, invasion and metastasis [10–13]. Lung cancer, one of the most deadly cancers, is regulated by many miRNAs [14]. Dozens of miRNAs, such as miR-143/145, miR-21, miR-34, and others, play essential roles in lung tumorigenesis by regulating critical oncogenes or tumor suppressors [15–17]. Recent studies have reported that miR-769-5p expression levels can predict overall survival in NSCLC [18]. However, the precise molecular mechanism by which miR-769-5p influences NSCLC progression remains largely unknown; thus, further investigations are required. In this study, we identified a new miRNA and signaling pathway in NSCLC, which may further elucidate the pathogenesis and provide promising therapeutic targets for NSCLC.

## RESULTS

### miR-769-5p was down-regulated and predicts poor prognosis in NSCLC

**Figure 1:**
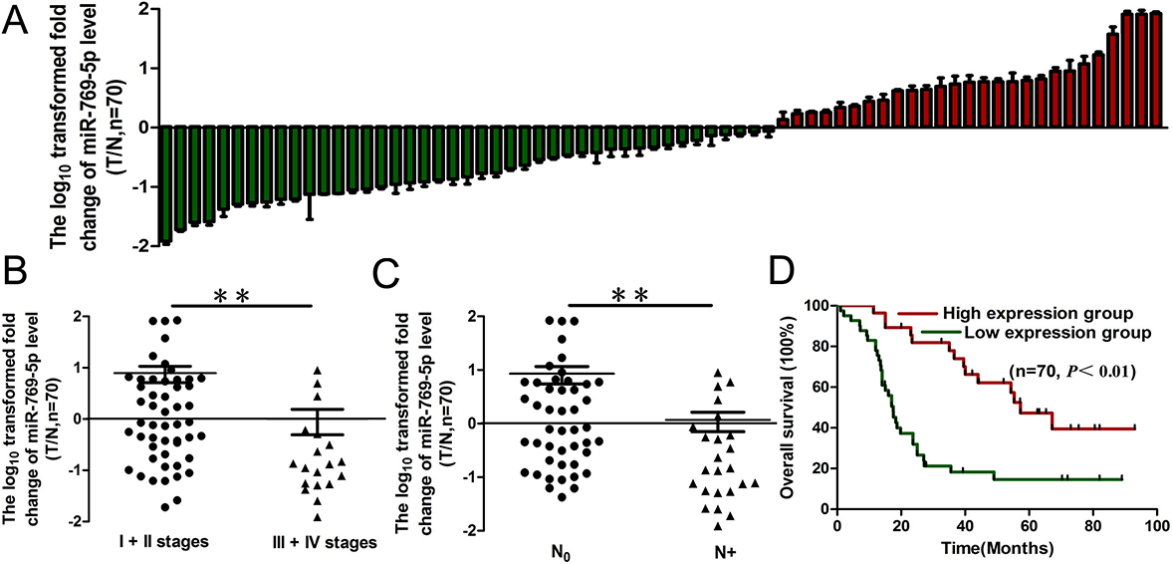
miR-769-5p expression in NSCLC and association with clinical factors (**A**) miR-769-5p levels were measured in 70 NSCLC samples and pair-matched lung tissues and normalized against an endogenous U6 RNA control by qRT-PCR analysis; (**B** and **C)** The expression of miR-769-5p in NSCLC was associated with clinical stages (B) and lymph node metastasis (C); (**D**) Kaplan–Meier curves depicting overall survival according to the expression of miR-769-5p. **p < 0.01.

To clarify the biological role of miR-769-5p in NSCLC tissues, we first measured the mature miR-769-5p level in 70 human primary lung tumors (NSCLC) and pair-matched lung tissues by qRT-PCR. For further analysis, all the samples were divided into a miRNA high expression group and low expression group according to the qRT-PCR results. The results showed that miR-769-5p expression in the tumors was significantly down-regulated (*p* < 0.05, Figure 1A, Table 1). miR-769-5p expression was not correlated with age, gender, tumor size, T stages, differentiation and local invasion (*p* > 0.05, Table 1), but it was correlated with clinical stage (*p* < 0.01, Figure 1B, Table 1) and lymph node metastasis (*p* < 0.01, Figure 1C, Table 1). Survival analysis revealed that down-regulated miR-769-5p was associated with poor prognosis in patients with non-small cell lung cancer (*p* < 0.01, Figure 1D).

**Table 1:** Correlation between miR-769-5p expression and clinicopathological characteristics in NSCLC patients

Characteristics	miR-769-5p expression level		
	Low (*n* = 43)	High (*n* = 27)	*P*
Gender			
Male	24 (55.8%)	14 (51.9%)	0.746
Female	19 (44.2%)	13 (49.1%)	
Age			
<60	20 (46.5%)	15 (55.6%)	0.461
≥60	23 (54.5%)	12 (44.4%)	
Tumor size			
≤3cm	27 (62.3%)	19 (70.3%)	0.515
>3cm	16 (37.7%)	8 (29.7%)	
Tumor stage			
T1 + T2	32 (76.2%)	22 (78.6%)	0.493
T3 + T4	11 (23.8%)	5 (21.4%)	
TNM stage			
I + II	27 65.1%)	24 (81.5%)	0.017
III + IV	16 (34.9%)	3 (18.5%)	
Histological grade			
Well/moderate	29 (67.4%)	15 (55.6%)	0.316
Poor/NS	14 (32.6%)	12 (44.4%)	
Lymph node metastasis			
Negative	24 (55.8%)	22 (81.5%)	0.028
Positive	19 (44.2%)	5 (18.5%)	

### miR-769-5p contributed to malignant phenotypes of NSCLC *in vitro*

**Figure 2:**
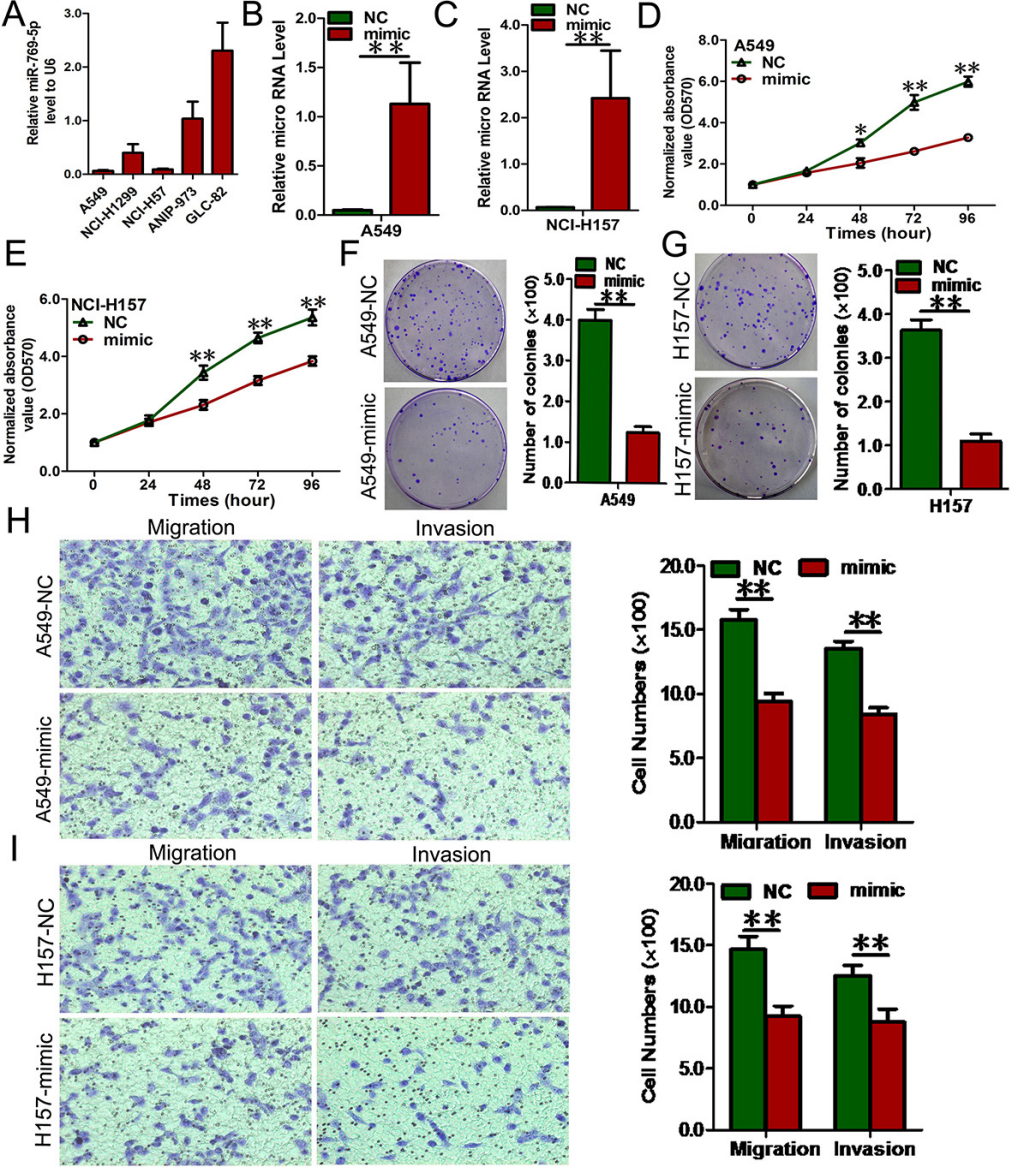
Elevated miR-769-5p inhibits cell proliferation, colony formation and migration (**A**) RNA level of miR-769-5p in 5 NSCLC cell lines; (**B** and **C**) Quantitative analysis of miR-769-5p level after the transfection of miR-769-5p mimic in A549 and H157 cell lines; (**D** and **E**) Cell growth curve was measured by MTS after the transfection of miR-769-5p mimic in A549 and H157 cell lines, and the OD 570 values were normalized to the start point (0 hour); (**F** and **G**) Representative images and quantitative analysis of colony formation was performed after the transfection of miR-769-5p mimic in A549 and H157 cell lines; (**H** and **I**) Representative images and quantitative analysis of transwell assays was performed after the transfection of miR-769-5p mimic in A549 and H157 cell lines. Data are presented as the mean value ± SD from triplicate experiments. *p < 0.05; **p < 0.01.

To elucidate the biological functions of miR-769-5p in NSCLC, we first examined miR-769-5p expression in 5 NSCLC cell lines, and the results showed that miR-769-5p was decreased in A549 and H157 cells but increased in A973 and GLC82 cells by qRT-PCR. (Figure 2A). We then transferred miR-769-5p mimics into A549 and H157 cell lines. qRT-PCR result confirmed that the miR-769-5p level increased significantly in cells transfected with the miRNA mimic (*p* < 0.01, Figure 2B, 2C). Transfection of A549 and H157 cells with the miR-769-5p mimic for 48, 72, and 96 hours significantly inhibited the cell viability (*p* < 0.05, Figure 2D, 2E). Moreover, colony formation, migration and invasion of the two cell lines were also suppressed as a result of miR-769-5p mimic transfection (*p* < 0.01, Figure 2F–2I).

**Figure 3:**
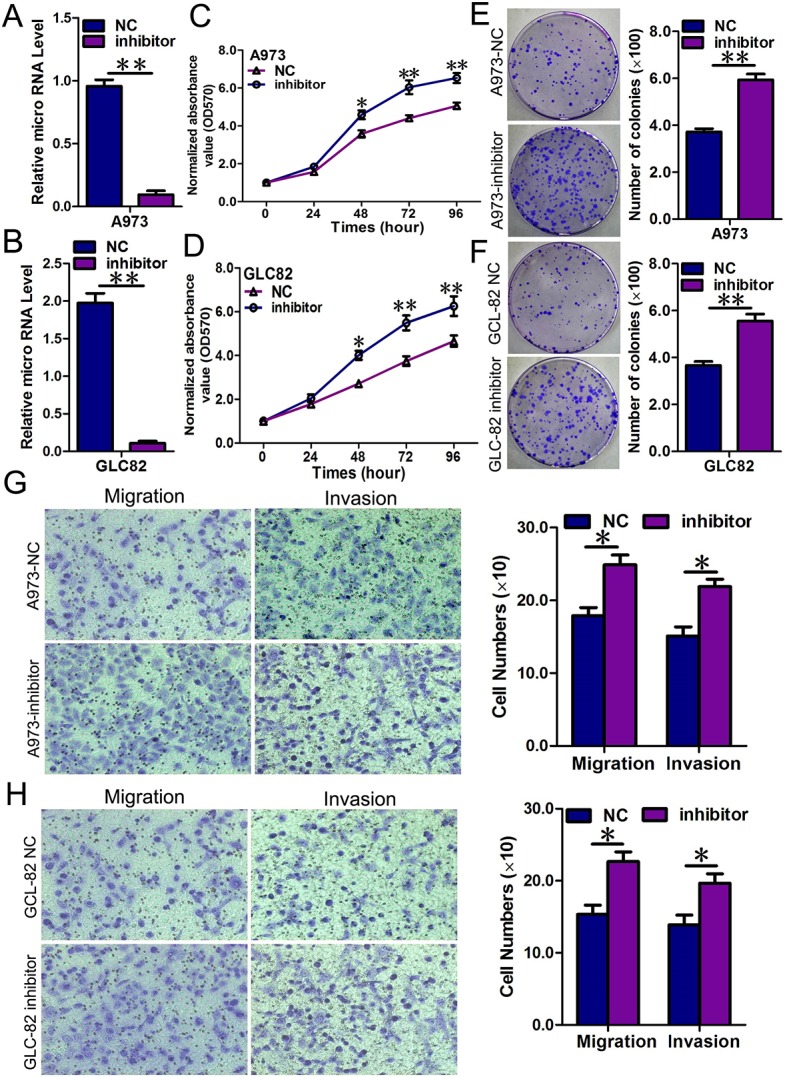
Repression of miR-769-5p in A973 and GLC82 cells significantly promoted cell growth, colony formation and migration; (**A** and **B**) Quantitative analysis of miR-769-5p level after the transfection of miR-769-5p inhibitor in A973 and GLC82 cell lines; (**C** and **D**) Cell growth curve was measured by MTS after the transfection of miR-769-5p inhibitor in A973 and GLC82 cell lines, and the OD 570 values were normalized to the start point (0 hour); (**E** and **F**) Representative images and quantitative analysis of colony formation was performed after the transfection of miR-769-5p inhibitor in A973 and GLC82 cell lines; (**G** and **H**) Representative images and quantitative analysis of transwell assays was performed after the transfection of miR-769-5p inhibitor in A973 and GLC82 cell lines. Data are presented as the mean value ± SD from triplicate experiments. *p < 0.05; **p < 0.01.

We also transfected NSCLC cells with inhibitors of miR-769-5p to confirm the opposite results of mimic transfection (*p* < 0.01, Figure 3A, 3B). As expected, down-regulation of miR-769-5p using inhibitors enhanced the malignant phenotype of A973 and GLC82 NSCLC cells *in vitro,* including cell growth (*p* < 0.05, Figure 3C, 3D), colony formation (*p* < 0.05, Figure 3E, 3F), cell migration and invasion (*p* < 0.05, Figure 3G, 3H). All the results using miR-769-5p mimics and inhibitors *in vitro* indicated that miR-769-5p inhibited proliferation, migration and invasion of NSCLC cells *in vitro*.

### TGFBR1 was a direct downstream target of miR-769-5p

**Figure 4:**
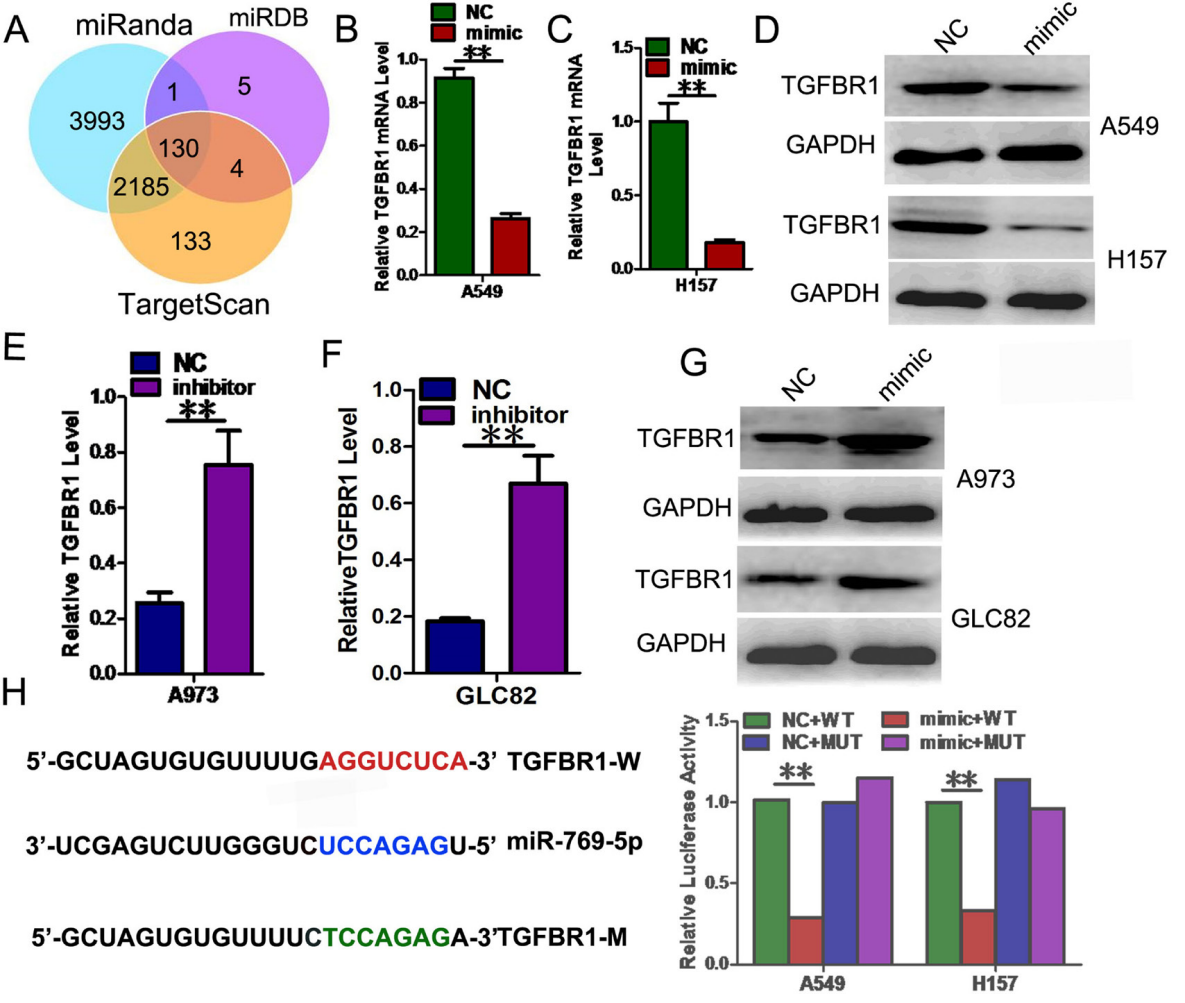
TGFBR1 is a direct target gene of miR-769-5p (**A**) TGFBR1 was identified as potential regulatory target of miR-769-5p by analysis of down-regulated genes using prediction tools; (**B**–**D)** The expression levels of the TGFBR1 mRNA and protein were measured by qRT-PCR and western blot analysis using GAPDH as the loading control after transfection of miR-769-5p mimic in A549 and H157 cell lines; (**E**–**G**) The expression levels of the TGFBR1 mRNA and protein were measured by qRT-PCR and western blot analysis using GAPDH as the loading control after transfection of miR-769-5p inhibitors in A973 and GLC82 cell lines; (**H**) Dual-luciferase reporter assay. The relative luciferase activity was normalized to the Renilla luciferase activity after co-transfection of cells with miR-769-5p mimic and pmiR-RB-REPORT™ construct containing the WT or MUT TGFBR1 3′-UTR region in A549 and H157 cell lines. Data are presented as the mean value ± SD from triplicate experiments. **p < 0.01.

To explore the mechanism by which miR-769-5p regulates NSCLC cell progression, we searched for potential regulatory targets of miR-769-5p using several bioinformatics methods, including TargetScan, miRDB, and miRanda (Figure 4A). In total, 130 genes were simultaneously predicted by the three databases, and transforming growth factor-β1 (TGFBR1) was detected as a candidate gene related to NSCLC based on its associated Gene Ontology (GO) terms and also harbors miR-769-5p binding sites, suggesting that TGFBR1 could be a potential target of miR-769-5p.

Transfected miR-769-5p mimics in cells strongly decreased the mRNA and protein levels of TGFBR1 (*p* < 0.01, Figure 4B–4D). However, the mRNA and protein levels of TGFBR1 were significantly increased after the transfection of miR-769-5p inhibitors (*p* < 0.01, Figure 4E–4G). We next performed dual-luciferase reporter assays to reveal the regulation of miR-769-5p to TGFBR1. The fragments containing the miR-769-5p binding sequence or mutated sequence from the 3′UTR regions of the TGFBR1 was cloned into the pmiR-RB-REPORT^™^ vector luciferease reporter, yielding pmiR-RB-REPORT^™^-TGFBR1-3′UTR and pmiR-RB-REPORT^™^-TGFBR1-3′UTR mut. These reporter constructs were co-tranfected with miR-769-5p mimic or miR-NC into A549 and H157 cells, and the luciferase activities were subsequently measured. The miR-769-5p mimic significantly suppressed the luciferase activity of pmiR-RB-REPORT™-TGFBR1-3′UTR (*p* < 0.01, Figure 4H), while miR-NC had no inhibitory effect on pmiR-RB-REPORT^™^-TGFBR1 -3′UTR. The miR-769-5p inhibition of pmiR-RB-REPORT™-TGFBR1 -3′UTR was sequence-specific because the luciferase activities of pmiR-RB-REPORT^™^-TGFBR1-mut did not reveal any reductions in the presence of miR-769-5p. Taken together, the results suggested that miR-769-5p can directly target the 3′-UTR of TGFBR1.

**Figure 5:**
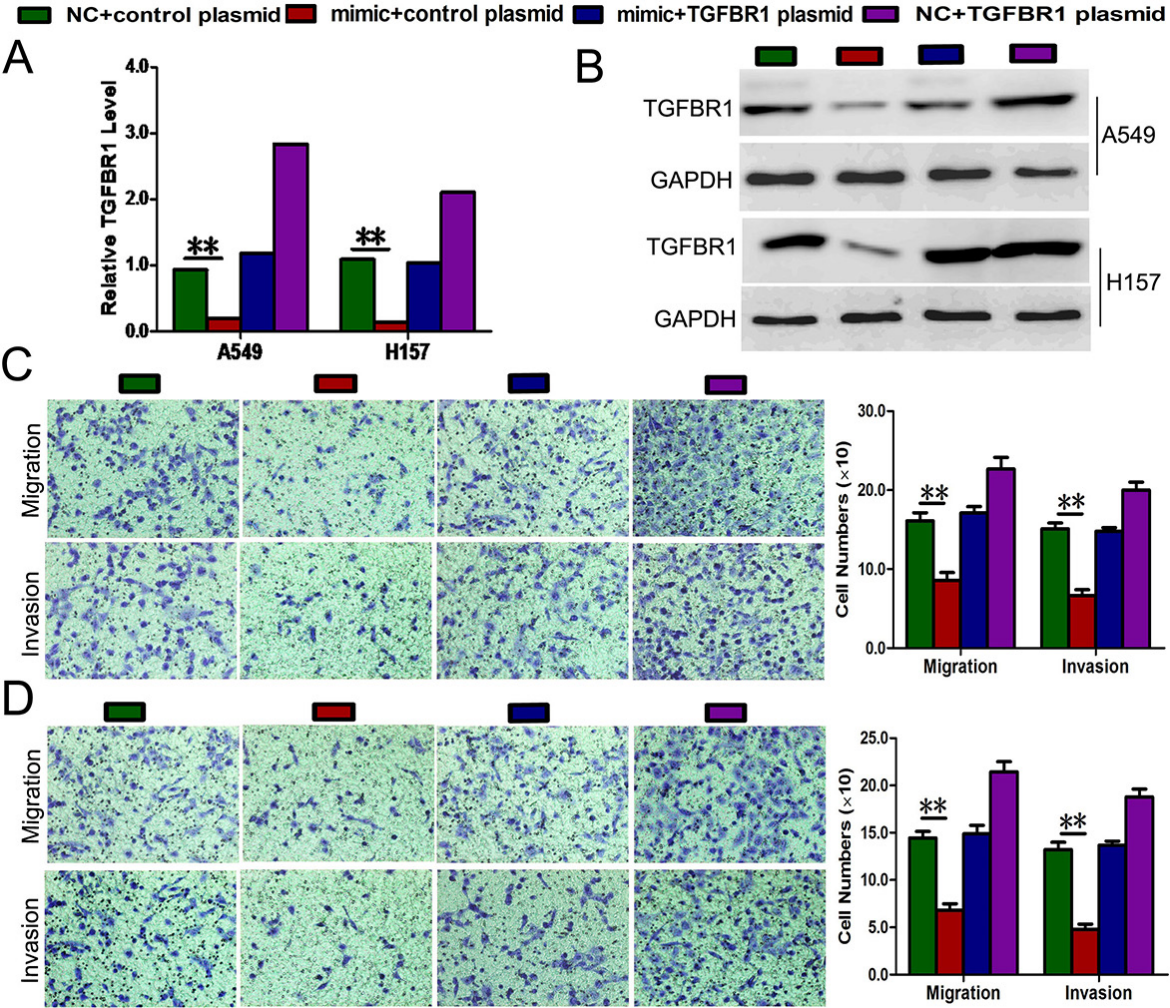
Rescue assays were further performed to confirm that TGFBR1 is a functional target of miR-769-5p (**A** and **B**) The mRNA and protein level of TGFBR1 in A549 and H157 cell lines with miR-769-5p mimic and pEGFP-C1 plasmid containing TGFBR1 CDS sequence. (**C** and **D**) Transwell assays of co-transfected cells with miR-769-5p mimic and TGFBR1 plasmid. Data are presented as the mean value ± SD from triplicate experiments. **p < 0.01.

Rescue experiments were performed to confirm that TGFBR1 is a functional target of miR-769-5p in A549 and H157 cells. TGFBR1 mRNA and proteins (endogenous) in the two cell lines were abolished by mimic transfection and recovered by transfection of both pEGFP-N1-TGFBR1 expression constructs (*p* < 0.01, Figure 5A, 5B). The results showed that migration and invasion induced by mimic transfection were reversed by transfection of both expression constructs (*p* < 0.01, Figure 5C, 5D). Moreover, Transfection of A549 and H157 cells with pEGFP-C1 plasmid containing TGFBR1 CDS sequence significantly promoted the cell viability cancer cell migration and invasion (p < 0.05, Figure 5C, 5D).

### miR-769-5p suppressed tumor growth and metastasis *in vivo*

**Figure 6:**
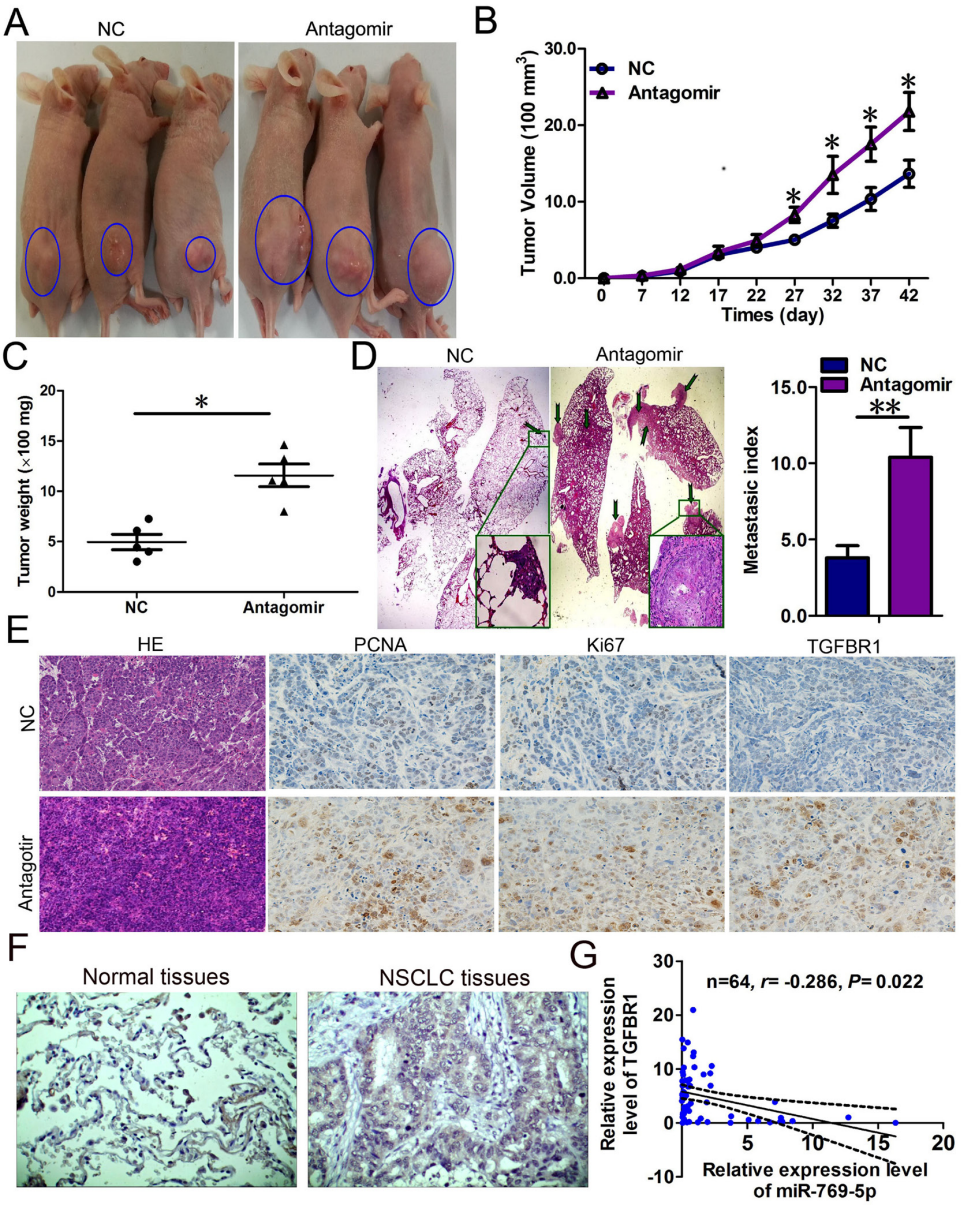
miR-769-5p suppressed tumor growth and metastasis *in vivo* and targeted TGFBR1 (**A**–**C**) The A973 cells were subcutaneously injected into nude mice to form solid and metastatic tumors and synchronously treated with miR-769-5p antagomir or miR antagomir NC (*n* = 5 for each group); (**D**) The number of metastatic nodules was observed and quantified in the lungs of mice treated with miR-769-5p antagomir or miR antagomir NC by the vein injection method. (**E**) Immunohistochemical staining of Ki67, PCNA and TGFBR1 in tumor tissues dissected from nude mice treated with miR-769-5p antagomir miR or antagomir NC. (**F**) Representative IHC images of TGFBR1 in NSCLCtissues and paired normal lung tissues are shown. (**G**) Spearman correlation analysis of the negative correlation between the expression of miR-769-5p and TGFBR1. *p < 0.05; **p < 0.01.

To confirm the tumor suppressor role of miR-769-5p *in vivo*, we established a BALB/c nude mouse tumorigenic and metastatic model using A973 cells. After 7 days, miR-769-5p antagomir or miR antagomir NC was directly injected into the implanted tumor and the tail vein every 5 days for seven times. The tumor volume was measured every 5 days until day 42. The tumor volume and weight of mice treated with the miR-769-5p antagomir were significantly increased relative to that of the miR antagomir NC (*p* < 0.05, Figure 6A–6C). Mice in the miR-769-5p antagomir group exhibited a considerably higher prevalence of metastasis in the lung than those in the miR antagomir NC group (*p* < 0.05, Figure 6D). The lungs had an average of 10 visible metastases in the miR-769-5p antagomir group, whereas visible metastasis was reduced by approximately 60% in the miR antagomir NC group (*p* < 0.01, Figure 6D). This result indicates that miR-769-5p significantly inhibits the tumorigenicity and metastasis of A973 cells in the nude mouse model. Additionally, the proliferative activities of tumor cells were assessed via immunohistochemical staining for Ki-67 and PCNA in FFPE tissues of xengraft tumors. The Ki-67 and PCNA staining intensities were decreased in the tumors from the miR-769-5p antagomir group (Figure 6E). TGFBR1 was found to be more intensely expressed in the miR-769-5p antagomir group than the miR antagomir NC group (Figure 6E). Moreover, an obvious increase in TGFBR1 protein expression was also observed between in NSCLC tissues and pair-matched lung tissues by immunohistochemistry staining (Figure 6F). The mRNA levels of TGFBR1 in NSCLC specimens were inversely correlated with the downregulation of miR-769-5p (*r* = –0.286, *p* < 0.05, Figure 6G).

## DISCUSSION

Lung carcinogenesis is a multistep process that results from activation of oncogenes and inactivation of tumor suppressor genes [19]. Although great progress has been made in early diagnosis and treatment methods in recent decades, the poor clinical outcome of this disease underscores a compelling need for further elucidation of the underlying molecular mechanisms of NSCLC [20]. Recently, numerous studies have indicated that many microRNAs have significant functions in carcinogenesis, with some correlated with clinical characteristics and prognosis outcomes [21]. Altered miRNA expression is commonly found in human carcinomas, e.g. NSCLC [22, 23]. Here, the role of miR-769-5p in lung carcinoma was evaluated, as well as the underlying molecular mechanisms.

In this study, the role of miR-769-5p in NSCLC progression was investigated. We first identified miR-769-5p as a novel anti-tumor miRNA in NSCLC that was significantly down-regulated in lung tumor tissue samples, and its expression levels were inversely correlated with clinical tumor stage and lymph node or distal metastasis by qPCR method. Recently, miRNAs linked to clinical tumor stages and lymph node metastasis of NSCLC, such as miR-10, miR-195, miR-338-3p, and miR-708-5p, have been identified [24–27]. miRNAs have also been extensively investigated as prognostic factors [28]. Additionally, our results showed that the high expression of miR-769-5p might be closely associated with advantageous patient overall survival. Zhou *et al.* also demonstrated that high expression of miR-574-5p in serum was an independent poor prognostic risk factor in patients with SCLC, as determined by multivariate Cox regression analysis of PFS and OS [29]. Therefore, our findings demonstrated that miR-769-5p may play a tumor-inhibiting role in NSCLC and prompted us to investigate its exact functions.

miRNA expression differences in tumor samples do not necessarily alter the function of tumor cells. Therefore, we further investigated the biological function of miR-769-5p *in vitro*. We showed that the miR-769-5p mimic suppressed the proliferation, colony formation and migration of NSCLC cells. Transfection of the miR-769-5p inhibitor had the opposite effects. Decreased miR-769-5p not only enhanced the cell viability but also promoted colony formation and cell migration. Importantly, data from the current study revealed that downregulation of miR-769-5p enhanced tumor growth and metastasis *in vivo* by delivery of antagonists into cancer cells and the tail vein of nude mice. Recent studies have shown that miRNAs act as tumor suppressors in NSCLC and other cancers [30, 31]. Joshi *et al.* also demonstrated that miR-148a might act as a tumor suppressor and inhibited migration and invasion of the A549 NSCLC cell line [32]. Yu *et al.* conducted an *in vitro* study of NSCLC cell lines and provided evidence that cell proliferation, migration and invasion were altered by transfection of miR-520a-3p [33]. Similarly, the role of miR-769-5p in inhibiting tumor growth and metastasis *in vivo* have been demonstrated in previous NSCLC studies [34, 35]. In gastric cancer, forced expression of miR-26b led to inhibition of GC cell migration and invasion *in vitro* and lung metastasis formation *in vivo* [36]. Conversely, miR-151-5p could promote tumor growth and lung metastasis of SC-M1 cells *in vivo* through down-regulation of p53 protein expression in NSCLC [37]. All these results support our findings that miR-769-5p functions as a tumor suppressor, and this miRNA may form the basis of new approaches for cancer therapy via its regulation of tumor regulatory properties.

To further elucidate the underlying mechanism of miR-769-5p on the development of NSCLC, we conducted an *in vitro* study using NSCLC cell lines and showed that direct targeting of TGFBR1 protein expression may mediate the tumor-suppressing function of miR-769-5p. TGFBR1 is an important element of the TGF-β/SMAD signaling pathway, which has emerged as a central mediator of cancer progression due to its capacity to regulate cell growth, differentiation and migration [38]. The TGF-β pathway controls a plethora of cellular responses and plays a crucial role in tumorigenesis as either a tumor promoter or suppressor [39]. For tumor-promoting effect, the TGF-β pathway stimulates cell invasion by inducing epithelial-mesenchymal transition (EMT) and eventually promoting metastasis, which has been extensively studied in multiple tumors [40–42]. TGF-β binding to TGFBR2 leads to phosphorylation and activation of TGFBR1 by TGFBR2. With the help of SARA, Smad2/3 are phosphorylated by TGFBR1. Then, they form a heterotrimeric complex with Smad4 and translocate into the nucleus to regulate gene transcription [43]. Yang *et al.* demonstrated that miR-140-5p can suppress hepatocellular carcinoma growth and metastasis by regulating TGFBR1 and FGF9 [44]. Studies have also shown that repression of TGFBR1 inhibited cell proliferation of lung cancer and cell migration and invasion of breast cancer [45, 46]. Immunohistochemical staining showed increased TGFBR1 staining intensities in xengraft tumors of the miR-769-5p antagomir group than in the NC group, indicating that tumor cell proliferation was increased when miR-769-5p was reduced. Consistent with previous reports, TGFBR1 was shown to function as a tumor oncogene in NSCLC and had lower expression in uterine cervix carcinoma [47, 48].

In summary, this investigation led to two novel observations. First, to our best knowledge, this is the first report showing that miR-769-5p is down-regulated in NSCLC, which is negatively correlated with the expression of TGFBR1, a newly discovered target in lung cancer. Second, miR-769-5p was shown to be independent prognostic factors in NSCLC. Thus, of particular interest, the miR-769-5p/TGFBR1 axis might represent a new molecular target for NSCLC treatment.

## MATERIALS AND METHODS

### Human NSCLC tissue samples

A total of 70 paired patient samples of primary lung cancer tissues and matched adjacent noncancerous tissues were obtained from the North China University of Science and Technology Affiliated People’s Hospital from 2013 to 2015. Histological characteristics of the samples are summarized in Table 1. Tissue samples were acquired during routine therapeutic surgery of patients who did not receive anti-tumor treatment. Written informed consent was obtained from patients for the use of their biological materials. This study was approved by the Ethics Committee of North China University of Science and Technology Affiliated People’s Hospital.

### Cell lines and cell culture

The human NSCLC cell lines A549, NCI-H1299, NCI-H157, ANIP-973, and GLC-82 were cultured in RPMI-1640 medium and human embryonic kidney (HEK293T) cells were maintained in DMEM supplemented with 10% fetal bovine serum at 37°C in a humidified air atmosphere containing 5% CO_2._

### miRNA transfection

All endogenous mature miRNA mimics, inhibitors and antagomirs were purchased from RiboBio (Guangzhou, China). Transient transfections of miRNA mimics/antagomirs/inhibitors were carried out using Lipofectamine 2000 (Invitrogen) according to the manufacturer’s protocol. Six hours following transfection, transfection media were replaced with culture media. All miRNA transfections were for 48 hours.

### Isolation of RNA, reverse transcription, and real-time PCR quantification

Total RNA was extracted from frozen tissues or cultured cells using TRIzol total RNA isolation reagent (Invitrogen) according to the manufacturer’s instructions. cDNA was synthesized from total RNA or purified small RNAs using gene-specific primers or random hexamers with the SUPERSCRIPT III Reverse Transcriptase Kit (Invitrogen), according to the manufacturer’s instructions. PCR was then performed using Taq polymerase (TaKaRa) with the specific primers for TGFBR1 (forward: 5′- TCGTCTGCATCTCACTCAT-3′, reverse: 5′-GATAAATCTCTGCCTCACG-3′) and GAPDH as an internal control (forward: 5′-TCTCTGCTCCTCCTGTTC-3′, reverse: 5′-GGTTGAGCACAGGGTACTTTATTGA-3′). MicroRNAs were detected with stem-loop primers purchased from RiboBio as described (Guangzhou RiboBio Co., Ltd). GAPDH and U6 small nucleolar RNA were used for normalization. qPCR was conducted using a QuantiTect SYBR Green PCR Kit (TaKaRa Bio Inc., Japan) on a StepOne Real-Time PCR System (Applied Biosystems, CA, USA). Relative expression levels were calculated using the 2^–ΔΔCt^ method (Bio-Rad CFX manager software 3.1).

### Plasmid construction

pDonR223-TGFBR1 plasmid carrying the human TGFBR1 gene was purchased from Changsha Axybio Bio-Tech Co., Ltd. (Changsha, China). The complete coding sequence of human TGFBR1 was amplified from the pDonR223-TGFBR1 plasmid. TGFBR1 PCR products and pEGFP-N1 plasmid were digested with XhoI and Hind III, and the fragments were purified and ligated with T4 DNA ligase. The ligated product was transformed into Top10 competent cells, and the positive clone was named pEGFP-N1-TGFBR1.

### Target prediction and luciferase reporter assays

Bioinformatics analysis was performed using specific programs: miRDB (http://www.mirdb.org/), miRanda (http://www.microrna.org) and TargetScan (http://www.targetscan.org/). The 3′-untranslated region (UTR) of human TGFBR1 was amplified from human genomic DNA and individually inserted into the pmiR-RB-REPORT™ (RiboBio, Guangzhou, China) using the XhoI and NotI sites. Similarly, the fragment of the TGFBR1 3′-UTR mutant was inserted into the pmiR-RB-REPORT™ control vector at the same sites. For reporter assays, A549 and H157 cells were co-transfected with wild-type (WT) reporter plasmid and miR-769-5p mimics. Firefly and Renilla luciferase activities were measured in cell lysates using the Dual-Luciferase Reporter Assay system. Luciferase activity was measured forty-eight hours post-transfection using the Dual-Glo luciferase reporter system according to the manufacturer’s instructions (Promega, Madison, WI, USA). Firefly luciferase units were normalized against Renilla luciferase units to control for transfection efficiency.

### Cell proliferation assay

Cell proliferation was evaluated by MTS (3-(4, 5-dimethylthiazol-2-yl) -5-(3-carboxymethoxyphenyl)-2-(4-sulfophenyl)-2H-tetazolium) according to the manufacturer’s instructions. Briefly, cells were plated into 96-well culture plates at an optimal density of 5 × 10^3^ cells/ml in 200 μl of culture medium per well. After 0–96 h of culture, MTS solution was added (20 μl/well) to each well and incubated at 37°C for 2 h. The optical density of each sample was immediately measured using a microplate reader (Bio-Rad, Hercules, CA, USA) at 570 nm.

### Colony formation assay

Cells were transfected with miR-769-5p mimic/miR mimic NC or miR-769-5p inhibitor/miR inhibitor NC. Twenty-four hours later, transfected cells were trypsinized, counted and replated at a density of 1 × 10^3^ cells/10 cm dish. Ten days later, colonies resulting from the surviving cells were fixed with 3.7% methanol, stained with 0.1% crystal violet and counted. Colonies containing at least 50 cells were scored. Each assay was performed in triplicate.

### Transwell migration/invasion assay

Cells were grown in RPMI 1640 medium containing 10% fetal bovine serum to ∼60% confluence and transfected with 50 nM miR-769-5p mimic, inhibitor or a negative control. After twenty-four hours, the cells were harvested by trypsinization and washed once with Hanks’ balanced salt solution (Invitrogen). The invasion assay was performed using 24-well Millicell hanging cell culture inserts with 8-μm PET membranes (Millipore) coated with BD Matrigel Basement Membrane Matrix separating the upper and the lower chambers. In the lower chamber, 500 μL of RPMI 1640 containing 10% FBS was added. Then, serum-free medium containing 5 × 10^4^ cells was added to the upper chamber for invasion assays. After twenty-four hours of incubation at 37 °C with 5% CO_2_, the number of cells that had migrated through the pores was quantified by counting 9 independent visual fields under the microscope (Olympus) using ×20 magnification, and cell morphology was observed by staining with 0.1% crystal violet for 20 min. Filters were washed thoroughly with 1× PBS. The migration assay is the same as the invasion assay except no Matrigel was used. Each experiment was performed at least three times.

### Western blot analysis

For western blot analyses, RIPA buffer containing protease inhibitors and phosphatase inhibitors (Roche) was used to prepare whole-cell lysates. Briefly, equal amounts of lysate were separated by SDS-polyacrylamide gel electrophoresis (SDS-PAGE) and then transferred to PVDF membranes (Millipore). After blocking with 5% bovine serum albumin (BSA), the membranes were probed with anti-TGFBR1 or anti-GAPDH (ab31013, ab9485, Abcam, Cambridge, UK), followed by incubation with a horseradish peroxidase-conjugated secondary antibody [goat-anti-mouse IgG (1:2000) and goat-anti-rabbit IgG (1:3000)]. Proteins were visualized by Image Reader LAS-4000 (Fujifilm) and analyzed by Multi Gauge V3.2 software.

### Tumorigenicity and metastasis assay *in vivo*

All animals received care in compliance with the “Guide for the Care and Use of Laboratory Animals” prepared by the Institute of Laboratory Animal Resources published by the National Institutes of Health and according to the Animal Experiment Guidelines of Samsung Biomedical Research Institute. The effect of miR-769-5p on the tumorigenic and metastatic potential of A973 cell was analyzed in orthotopic and systemic metastasis *in vivo* models. For the subcutaneous and metastatic models, 3–4-week-old BALB/c nude mice were injected subcutaneously in the right hip and the tail vein with A973 cells (1 × 10^6^ in 100 μl of HBSS). After 7 days, the transplanted and metastatic nude mice were randomly divided into two groups, respectively (*n =* 5 each). miR-769-5p antagomir or miR antagomir NC (NC) was directly injected into the implanted tumor and the tail vein at a dose of 1 nmol (in 50 μL phosphate-buffered saline) per mouse every 5 days for seven times. The tumor size was monitored by measuring the length (L) and width (W) with calipers every 5 day, and the volumes were calculated using the following formula: (L × W^2^)/2. Mice were killed by cervical dislocation on day 42, and the tumors were excised and snap-frozen for protein and RNA extraction.

### Evaluation of immunohistochemical staining

Immunohistochemistry staining was performed on 4 μm-thick slices following the EnVision two-step procedure of the Dako REAL™ Envision™ Detection System (Dako). Slides were incubated with primary antibodies, including PCNA (1:100, ab18197, Abcam), Ki-67 (1:50, ab833, Abcam) and TGFBR1 (1:50), followed by incubation with HRP-labeled secondary antibody and were visualized with diaminobenzidine. The expression status of TGFBR1 in the cytoplasm was determined by the product score of the average percentage and intensity of positive cells under 5 random high-power fields. The score for percentage is as follows: <5% (0), 5%–25% (1), 25%–50% (2), 50%–75% (3), and >75% (4); the score for intensity is as follows: no staining (0), light brown (1), brown (2), and dark brown (3). For TGFBR1, a score of 0 and ≥1 was defined as negative and positive, respectively.

### Statistical analysis

All values in this study are expressed as the mean ± SD, and all error bars represent the standard deviation of the mean. Student’s *t* test, χ2 test and repeated measures ANOVA were used to determine significance. The log-rank test was used to analyze the effect of clinical variables and miRNAs on patients’ overwell surviall (OS). All statistical tests were two-sided. *p* < 0.05 was considered statistically significant. Data were imaged with GraphPad Prism 5 software. Statistical analyses were performed using SPSS 16.0 software (SPSS Inc, USA).
